# The surface properties of nanoparticles determine the agglomeration state and the size of the particles under physiological conditions

**DOI:** 10.3762/bjnano.5.188

**Published:** 2014-10-15

**Authors:** Christoph Bantz, Olga Koshkina, Thomas Lang, Hans-Joachim Galla, C James Kirkpatrick, Roland H Stauber, Michael Maskos

**Affiliations:** 1 Fraunhofer ICT-IMM, Carl-Zeiss-Straße 18–20, 55129 Mainz, Germany; 2 BAM Federal Institute for Materials Research and Testing, Unter den Eichen 87, 12205 Berlin, Germany; 3 Institute of Biochemistry, Westfälische Wilhelms Universität, Wilhelm-Klemm-Str. 2, 48149 Münster, Germany; 4 Institute of Pathology, University Medical Center of Mainz, Langenbeckstraße 1, 55101 Mainz, Germany; 5 Molecular and Cellular Oncology/Mainz Screening Center, University Medical Center of Mainz, Langenbeckstraße 1, 55101 Mainz, Germany

**Keywords:** nanomaterial characterization, physiological conditions, surface properties, silica nanoparticles, siloxane nanoparticles

## Abstract

Due to the recent widespread application of nanomaterials to biological systems, a careful consideration of their physiological impact is required. This demands an understanding of the complex processes at the bio–nano interface. Therefore, a comprehensive and accurate characterization of the material under physiological conditions is crucial to correlate the observed biological impact with defined colloidal properties. As promising candidates for biomedical applications, two SiO_2_-based nanomaterial systems were chosen for extensive size characterization to investigate the agglomeration behavior under physiological conditions. To combine the benefits of different characterization techniques and to compensate for their respective drawbacks, transmission electron microscopy, dynamic light scattering and asymmetric flow field-flow fractionation were applied. The investigated particle systems were (i) negatively charged silica particles and (ii) poly(organosiloxane) particles offering variable surface modification opportunities (positively charged, polymer coated). It is shown that the surface properties primarily determine the agglomeration state of the particles and therefore their effective size, especially under physiological conditions. Thus, the biological identity of a nanomaterial is clearly influenced by differentiating surface properties.

## Introduction

### Accurate characterization of nanomaterials for use in biological environments

Since the huge surface area to volume ratio of nanoparticles (NPs) provides an enormous reactive interface between the particle and its local environment, the properties of materials with identical chemical composition can differ significantly depending on whether the compound is applied as bulk material or as nanomaterial (NM). The presence of NMs in daily life products, the need for a health risk estimation that arises from this presence and potential applications in the field of biomedicine demand an investigation of the bio–nano interface [1–4].

As an essential part of assessing medical benefits or potential harms of nanomaterials, a comprehensive and accurate characterization of the material is inevitable to be able to clearly correlate the observed biological impact with defined colloidal properties [5–7]. There is a multitude of crucial NP properties that could be addressed in such a characterization, for example, size, size distribution, shape, crystal structure, chemical composition, surface area, functional groups, charge or porosity [8]. However, since an exhaustive characterization of nanomaterials is complex and therefore both time- and cost-consuming, a customized characterization focusing on the objective of the study is recommended [9]. To ensure comparability of experiments, standardization of characterization methods must be developed and applied [10].

Since nanomaterials gain more and more importance as subjects of in vitro and even in vivo studies, a proper characterization of the NPs especially in biologically relevant media becomes crucial because pristine colloidal properties can alter therein. This is particularly valid in the presence of proteins [11] because NPs tend to form a protein corona [12–13] that is considered to mediate cellular responses [14] and uptake pathways [15–16]. Depending on the nature of colloidal stabilization, the formation of a protein corona and/or even the conditions of physiological salinity can furthermore induce agglomeration. This affects particle characteristics especially with respect to particle size and size distribution, which can influence the biodistribution, circulation time, intracellular trafficking, clearance or uptake mechanism [17].

Within this paper, we present the size characterization of two SiO_2_-based nanomaterials: silica nanoparticles that are electrostatically stabilized by negative surface charges and poly(organosiloxane) nanoparticles that were modified to carry positive charges and/or a polymer shell. We demonstrate a combination of carefully chosen characterization techniques, that is, transmission electron microscopy (TEM), multiangle dynamic light scattering (DLS) and asymmetric flow field-flow fractionation (AF-FFF). By combining the strengths of each of the individual techniques, their drawbacks are compensated for and a comprehensive characterization in physiological media is possible. As a result, it is shown that differences in the agglomeration state and therefore in size were observed under physiological conditions both in presence and in absence of serum proteins. This is especially relevant with regard to in vitro studies.

### Characterization techniques

As mentioned, only by combining different characterization techniques can one compensate for the drawbacks of each technique. This section gives a short introduction into each of the techniques that were applied in this study. Special emphasis is placed on the advantages and disadvantages [9,18–19].

**TEM:** Transmission electron microscopy (TEM) is a widely applied technique that uses a focused electron beam to image nanometer-sized structures [20]. Within a very short time, TEM gives at least a quantitative overview over the most important properties of the sample (shape, size and size distribution). However, analysis of TEM data is always based on single-particle measurements. For a precise and reliable quantification of the mentioned characteristics from TEM images, care must be taken in the statistical evaluation of TEM data. A second disadvantage arises from the fact that TEM requires vacuum conditions to reach a reasonable resolution. Thus, particulate samples cannot be investigated in dispersed state and are commonly prepared by dry preparation. This involves placing a droplet of the particle dispersion on a carbon- or polymer-coated copper grid then the solvent is allowed to evaporate. However, this procedure leads to preparation artifacts that mainly pertain to the arrangement of the particles. Therefore, standard TEM is useful to obtain information about the pristine state of particles but does not provide information about the hydrodynamic properties of a sample, for example, its agglomeration state in a physiologically relevant medium [18,21].

The latter question can only be addressed by cryogenic transmission electron microscopy (cryo-TEM). For sample preparation, the method of plunge freezing is applied here: The vitrification of aqueous samples in liquid propane or ethane prevents the water film that covers the grid from crystallizing and leads to a fixation of the state in solution. The investigation under frozen–hydrated conditions allows the visualization of a particulate sample under physiological conditions in a state that is as close as possible to its native state in solution [21–23]. However, as cryo-TEM investigations require an experienced operator and are both cost- and time-consuming, the method is far from becoming a standard technique.

**DLS:** In dynamic light scattering (DLS), the fluctuations of the intensity of light scattered by a colloidal dispersion are observed over time and the analysis of the self-correlated data yields information about the hydrodynamic radius (*R*_h_) of the sample. The fluctuations in scattering intensity initially originate from the Brownian motion of the particles [24–25]. For data analysis, these fluctuations are transferred to the autocorrelation function, *g*_1_(τ), which is a mathematical description of how the scattering signal at a given time *t* is related to the signal at a later time, *t* + τ. Information about the self-diffusion coefficient (*D*_s_) is gained from the decay of the autocorrelation function by applying the so-called Siegert relation. By subsequent application of the Stokes–Einstein equation, the hydrodynamic size can be calculated from *D*_s_ [26].

DLS is a very powerful characterization technique as it yields absolute values for an ensemble of particles. Additionally, it allows for the investigation of the properties of the particles in solution, even under physiologically relevant conditions. Therefore, a multitude of DLS setups is commercially available today, most of them operating at one single scattering angle. Nevertheless, there is a clear benefit of using a multiangle setup for the investigation of complex samples and/or complex questions, such as the investigation of the agglomeration behavior of colloids under physiological conditions. While addressing questions of such complexity, the necessity for a high level of expertise in light scattering data acquisition, evaluation, and interpretation arises from the fact that several challenging issues can occur [27]. First, in the case of samples being not truly monodisperse, one must extrapolate *q^2^* → 0 to reveal the true value of the inverse z-average of the sphere-equivalent hydrodynamic radius, 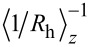. As the scattering vector, *q*, mainly depends on the scattering angle, θ, this requires angular-dependent measurements. Otherwise (when using a single-angle setup), only apparent size values are obtained and with increasing polydispersity, these can differ significantly from the true size [18]. Second, as the scattering intensity scales with the sixth power of particle size (*I* ~ *R*^6^), the detection of smaller particles is hampered in the presence of larger particles. This necessitates the removal of dust particles (if the data are not post-processed) and makes the separation of fractions of multimodal samples (e.g., agglomerates alongside with non-agglomerated particles) a very sophisticated task [28]. Third, the µ_2_ value that results from cumulant analysis allows for an estimation of the width of the distribution of the self-diffusion coefficients and is generally used to assess polydispersity from DLS data. As a convention, the μ_2_ values at a scattering angle of 90° are usually given. Roughly estimated, samples with μ_2_ values smaller than 0.05 are considered to be monodisperse, whereas samples with μ_2_ values greater than 0.2 are considered to be polydisperse [29]. Calculations of size distributions and polydispersity values from μ_2_ should be treated with care since non-Gaussian size distributions as well as disturbances can distort the derived distributions. When measuring in deionized water, such a disturbance could originate from long-range, inter-particle interactions leading to artifacts in the angular dependency of the apparent diffusion coefficients. This is the so-called structure factor, *S*(*q*) [30–31].

**AF-FFF:** Asymmetric flow field-flow fractionation (AF-FFF) describes a quasi-chromatographic, semi-preparative particle separation method and is the best known and best established representative of the versatile field-flow fractionation (FFF) family [32–36]. As hydrodynamic sizes and size distributions of particles ranging from 1 nm to several µm can be analyzed, AF-FFF is a powerful tool for sample separation and size characterization of nanoparticles both in aqueous and in organic solution. The separation is realized by a ribbon-like fractionation channel in which the sample is transported by the carrier liquid that generates a lamellar flow profile. This axial flow is superposed by a homogeneous drainage of the carrier liquid at one channel border, which induces a drift of the sample towards the accumulation membrane where slow axial flow velocities are present. Retention will occur according to the average distance of the sample to the accumulation wall, which is determined by the cross flow induced drift and the size-dependent diffusion coefficient of the particles. Thus, particles separate according to their hydrodynamic properties [37]. Typically, the hydrodynamic radius can be determined by measuring the retention time of size standards and applying the calibration data to the elugram of the sample [38]. Since particle–particle interactions as well as particle–membrane interactions cannot be neglected, size determination in AF-FFF is not absolute [39].

The mentioned interactions are strongly affected by the concentration and the type of the electrolyte that is added to the eluate. When increasing the salinity of the dispersant, electrostatic repulsions of colloids are reduced by decreasing the Debye screening length, κ^−1^, as described by the DLVO theory [40]. Additionally, specific ion phenomena arise from different polarizabilities and Pearson acidities of the ions, leading to a variation of ion–solvent interactions [39]. Specific ion properties are summarized in the Hofmeister or lyotropic series [41–42], which orders ions according to the magnitude of their destabilizing effect on colloidal dispersions. Nevertheless, small amounts of salt should be added to the eluate in AF-FFF to minimize electrostatic repulsion [43]. For application in the fields of the bio-nano sciences, physiological salt contents are of special interest. However, little success was achieved when operating AF-FFF setups at 150 mM salinity. Practically, an upper concentration limit of 50 mM NaCl was found [44]. This can be attributed to the fact that hydrophobic interactions are present at higher salt concentrations, leading to reversible or irreversible sample adsorption onto the membrane. Peak broadening and peak asymmetry are observed consecutively [45] as well as a loss in the recovery rate [46–47]. By coupling of a variety of online detectors to the FFF instrument, an extensive sample characterization is possible, providing a comprehensive analysis with emphasis on single fractions. Relevant online detectors are based on fluorescence detection and UV absorbance to determine retention times and for an appropriate concentration determination. Also, absolute size characterization by static light scattering (multiangle laser light scattering, MALLS), dynamic light scattering [48–49], or mass spectrometry (inductively coupled plasma mass spectrometry, ICP-MS) [50] can be applied.

### Silica and poly(organosiloxane) nanoparticles

Although both silica and poly(organosiloxane) (POS) nanoparticles are based on silicon dioxide (SiO_2_) as building blocks and are chemically closely related, the two systems exhibit different properties. These different properties result in different fields of applications. “Simple” (non-modified) silica particles have been widely used for decades as additives in the tire and construction industry, in cosmetics, and even in food [51]. Furthermore, both silica and siloxane particles are promising candidates for applications in the field of nanomedicine. In particular, the poly(organosiloxane) particles offer high versatility and flexibility with regard to architecture, composition and functionalization. Hence, they are highly suitable for tailored biomedical applications, for example, as drug carrier systems, as agents in hyperthermia, or as contrast agents for magnetic resonance imaging (MRI) [52–53].

**Silica nanoparticles:** As most of the common crystalline SiO_2_ particles are not in the nanometer-size region, we will focus on amorphous silica nanoparticles (aSNPs) in the following section. Based on their preparation procedure, aSNPs can be divided into three main subclasses [54]:

1. Silica sols (also called “colloidal silica”) are manufactured on the industrial scale by acidification of waterglass (aqueous solutions of alkali silicates, e.g., Na_2_SiO_3_, Na_4_SiO_4_). Typically, this neutralization is performed by using ion exchange resins where particle sizes of around 5 to 500 nm in diameter can be realized [55–57].

2. Pyrogenic silica (also referred to as “fumed silica”) is produced on the industrial scale as well. By pyrolysis of, for example, silicon tetrachloride, primary particles with diameters less than 50 nm are obtained. These merge irreversibly into aggregates with diameters between 250 nm and a few tens of micrometers [58–59].

3. The third synthesis route involves the hydrolysis and condensation of silicic acid esters (mostly tetraethyl orthosilicate, TEOS) under basic alcoholic conditions. It was first described by W. Stöber in 1968 [60] and gives well defined particles with sizes of around 20 to 5,000 nm. Numerous improvements allow for the synthesis of core–shell particles, of dye-labelled particles, or of particles with defined surface functionalities [61–64]. Furthermore, the reaction principle of the Stöber synthesis is also applied for the preparation of mesoporous silica nanoparticles (MSN) [65].

With respect to particle size distributions, silica sols and Stöber particles are typically relatively monodisperse. Standard deviations are found in the range of 15 to 30% for silica sols and in the range of 5 to 15% for Stöber particles. For both methods, this depends on the synthesis conditions and on the particle size. In contrast, pyrogenic silica particles exhibit broad size distributions. Due to the differences in the preparation procedures, the three silica particle types exhibit different properties beyond the realizable particle size and particle size distribution, for example, with respect to density and surface porosity [54,56,58–59].

Silica nanoparticles are already widely investigated. Therefore, they are an ideal reference system to demonstrate the potential of the combination of characterization techniques that we have chosen. Parts of the characterization data acquired within this study were also used as the basis for nanotoxicology studies [51]. As reported there, NexSil20 is a commercially available, colloidal silica without surface modification. Keeping in mind its application to nanotoxicology studies, this system was chosen due to the fact that colloidal silica is used for most real life applications of silica nanoparticles.

**Poly(organosiloxane) nanoparticles:** Poly(organosiloxane) NPs are synthesized in an aqueous dispersion by co-condensation of mixtures of dialkyldialkoxysilanes and alkyltrialkoxysilanes in the presence of a surfactant. By sequential addition of mixtures with different ratios of bi- to tri-functional monomers, different crosslinking densities can be realized and the formation of systems with core–shell architecture is possible. By introducing monomers with functional groups, each of the structural elements (core, shell or one of multiple shells) can be functionalized independently [66–68]. The addition of a chloromethylphenyl co-monomer for instance allows for selective fluorescence labeling of the core [69–70]. To obtain an unmodified, hydrophobic surface, the reactive residues on the shell are saturated with a trialkylalkoxysilane. In contrast, the application of appropriately functionalized monomers yields either an unreactive (but functionalized) particle surface, or a surface which is covered with reactive groups that can be further converted in subsequent reactions (e.g., to achieve water solubility) [53].

Particle diameters in the range of 15 to 150 nm with polydispersity values comparable to those of Stöber silica particles are possible. Due to the incorporation of organic residues into the siloxane network, the poly(organosiloxane) NPs have a lower density than Stöber particles (typical values on the order of 1.1 to 1.5 g/cm^3^). Since the synthesis allows for a very selective functionalization of any particle compartment, POS particles show higher versatility with respect to the variety of possible functionalizations. This makes it possible to change the polarity of the particles. Due to that, the poly(organosiloxane) NPs are a suitable system for a very interesting application in the field of nanotoxicology research: the investigation of the interaction of nanoparticles with the components of the alveolar surfactant film [71–73]. As the lung surfactant is the first barrier that inhaled nanoparticles encounter after passing the respiratory tract [74], interactions with this barrier are crucial in inhalation toxicology.

The preparation of the poly(organosiloxane) particles that we report on is based on previously described syntheses by our group [66,69]. These procedures were extended as follows [75]: First, core–shell particles with a dye-labelled core were synthesized by using the basic synthesis route. The shell was functionalized with amine groups (–NH_2_) by addition of *N*-(6-aminohexyl)aminopropyltrimethoxysilane (AHAPS) to the monomer mixture for the shell. Reactive residues on the surface were saturated with ethoxytrimethylsilane by application of the usual two-stage endcapping procedure. Due to the polar end groups in the shell, the particles are soluble in polar organic solvents and the second stage of the endcapping procedure was performed in methanol. Subsequently, amine functionalized poly(organosiloxane) nanoparticles POS-NH_2_ in water were obtained after dialysis in 2-(*N*-morpholino)ethanesulfonic acid (MES) buffer and water.

**Scheme 1:**
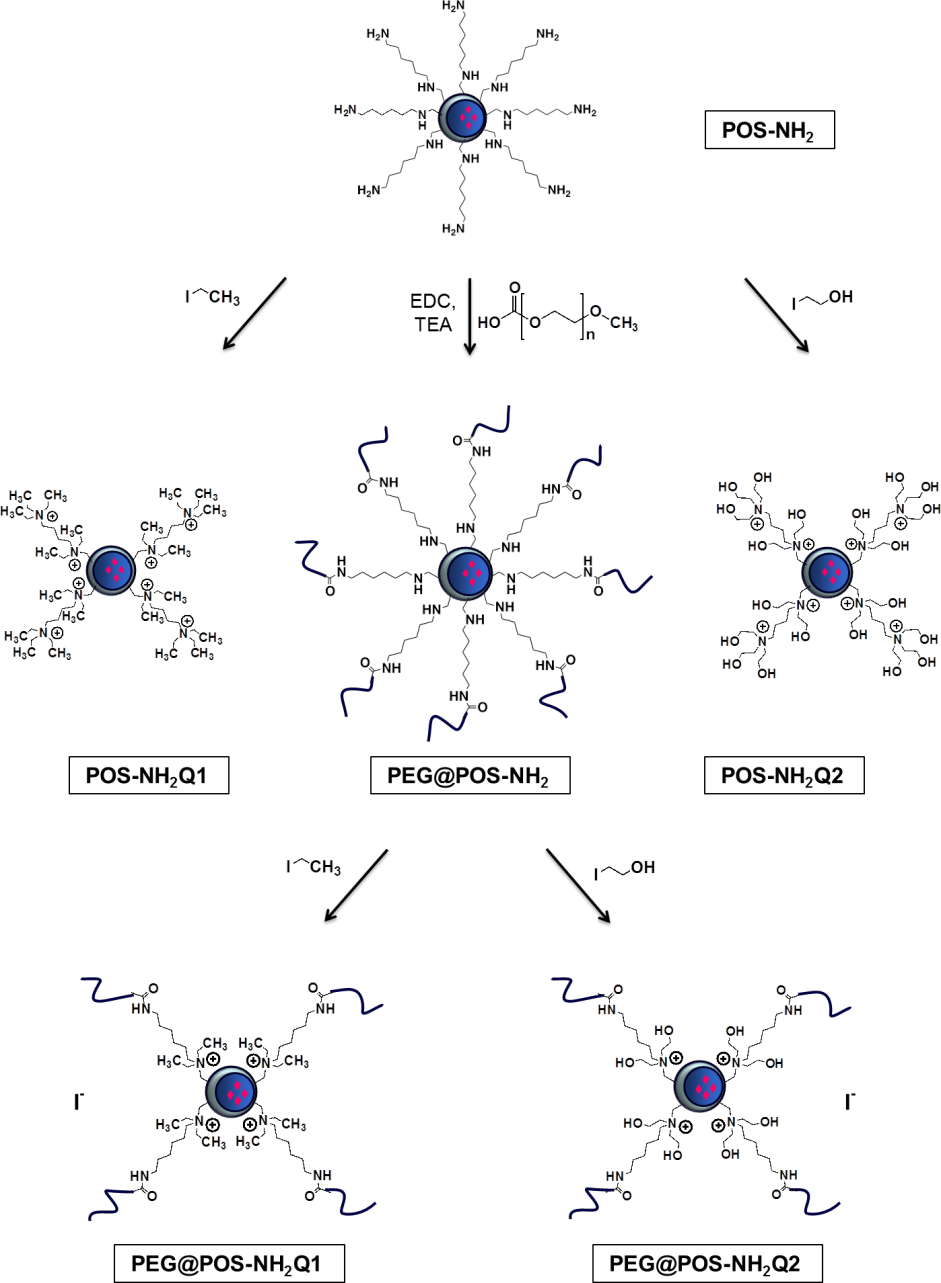
Schematic illustration of the synthesis routes for the preparation of quaternized and/or PEGylated poly(organosiloxane) nanoparticles starting from AHAPS-modified particles.

As shown in Scheme 1, the surface of the particles can be loaded with physical charges by alkylation of the amine groups to guarantee the positive charge of the surface independent from salt conditions. For this purpose, POS-NH_2_ nanoparticles in methanol were reacted with ethyl iodide and 2-iodoethanol yielding the quaternized particles POS-NH_2_Q1 and POS-NH_2_Q2. Alternatively, the amine groups can be used for grafting reactions with polymers in a “grafting onto” approach leading to a shift of the stabilization mechanism towards steric stabilization. Hence, a carboxy (–COOH) terminated poly(ethylene glycol) (PEG, molecular weight ca. 2 kDa) was reacted through a coupling reaction mediated by 1-ethyl-3-(3-dimethylaminopropyl)carbodiimide (EDC) in methanol (PEG@POS-NH_2_). Finally, the combination of both modification steps was achieved, which yields sterically stabilized particles with additional charges within the polymer shell (PEG@POS-NH_2_Q1 and PEG@POS-NH_2_Q2). To accomplish this, the POS-NH_2_ nanoparticles were first PEGylated and then the remaining ammonium groups were quaternized. As all of the described modification steps are carried out in methanol, a dialysis step is needed at the end of each synthesis to transfer the particles into water. The possibilities for modifications of the AHAPS functionalized poly(organosiloxane) nanoparticles are summarized by a schematic illustration of the synthesis routes in Scheme 1.

## Results and Discussion

### Comparison of the agglomeration behavior of silica and siloxane nanoparticles

In the following, the results of extensive size characterization of the synthesized siloxane particles under non-physiological as well as under physiological conditions are described and compared to the data obtained for the negatively charged silica NPs. Such studies are important as the utilization of a well-characterized material with defined properties is necessary to be able to correctly interpret the results of any study on the physiological impact of nanomaterials (e.g., in nanotoxicology or with regard to potential biomedical applications).

#### Characterization under non-physiological conditions

**Figure 1:**
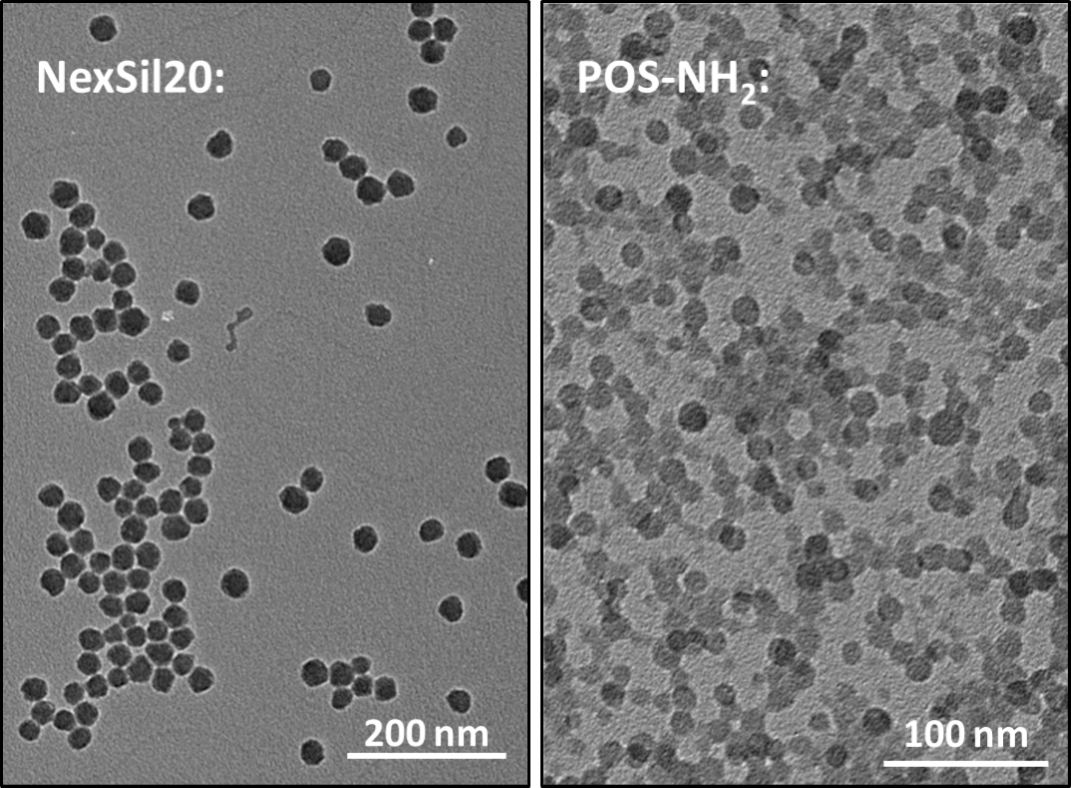
TEM micrographs of NexSil20 and POS-NH_2_ nanoparticles after dry preparation from an aqueous dispersion.

Both the silica as well as the poly(organosiloxane) nanoparticles were first analyzed by TEM to get a quick overview of the properties of the samples (Figure 1). The TEM pictures show a nearly spherical shape for both particle types. By manual image analysis, the radius of the silica particles was estimated to be 15.4 ± 2.2 nm (±14%, analysis of over 100 particles) and the radius of the POS particles was estimated to be 7.8 ± 1.3 nm (±17%, analysis of over 60 particles). For a more reliable size determination from TEM images, more sophisticated analysis routines should be applied. Specifically, this should include the analysis of more particles (>500) and, if possible, an automated image analysis to increase reproducibility [76]. The silica particles are found either as single particles or in smaller groups, whereas the siloxane particles are found in large clusters or arranged in layers. In contrast, the characterization of the hydrodynamic properties in the following does not show large agglomerates for these samples under non-physiological conditions. This provides evidence that dry preparation of colloidal samples induces preparation artifacts. During all of the modification steps of the POS-NH_2_ particles, organic or polymeric materials were introduced. Due to the lower contrast of carbon atoms compared to silicon, none of these modifications can be visualized by TEM, at least without the application of contrast agents. As staining procedures might cause additional artifacts and furthermore also lead to an increase in preparation time, no staining was applied here and for the POS particles the TEM investigation is only useful at the first stage of the modification procedure.

To compensate for the mentioned drawbacks of the TEM data evaluation, angular-dependent DLS measurements were performed. The results are summarized in Table 1. To evaluate the DLS data, bi-exponential fitting functions were primarily used. Additionally, cumulant analyses were performed at scattering angles of 90° to derive μ_2_ values as a qualitative measure for polydispersity. In the third column, the hydrodynamic radii that were obtained for measurements in deionized water are shown. One potential problem of measuring colloids in deionized water was already described above. That is, the inter-particle interactions can cause artifacts in the angular dependency of the apparent diffusion coefficients [26]. Additionally, ionic impurities and dissolved carbon dioxide cause the presence of ions in minor amounts leading to the fact that salinity is purely defined. Therefore, the addition of small amounts of salt, for example, 5 mM NaBr as chosen here, does not disturb the measurement, but rather promotes constant salinity.

**Table 1:** Hydrodynamic radius, *R*_h_, and μ_2_ values in different media for the samples discussed in this publication. NexSil20 NPs are commercial silica nanoparticles, POS-NH_2_ NPs are amine-modified poly(organosiloxane) particles at different modification stages: bare, quaternized, PEGylated and PEGylated + quaternized. DLS measurements were performed with an ALV multiangle setup, all samples were filtered after mixing of the components, and radii were determined either by applying a biexponential fitting function (non-agglomerated samples) or by conducting a multicomponent analysis in the cases where proteins were present [28]. DLS analysis of the pure protein mixture in RPMI cell culture medium (RPMI + 5% FCS) yields an average hydrodynamic radius of 9.2 nm (μ_2_: 0.16). Furthermore, the results of zeta potential (ZP) measurements in low salt containing water are shown; these measurements were performed by using a Malvern Zetasizer.

	Water (5 mM NaBr)			RPMI		RPMI + 5% FCS	
	ZP / mV	*R*_h_ / nm	μ_2_(90°)	*R*_h_ / nm	μ_2_(90°)	*R*_h_ / nm	μ_2_(90°)

NexSil20	-40	17.1	0.11	17.6	0.09	91	0.11
POS–NH_2_	31	13.5	0.11	prec.^a^		agglom.^b^ (128)	
POS–NH_2_Q1	27	15.6	0.29	prec.^a^		agglom.^b^ (219)	
POS–NH_2_Q2	32	15.8	0.27	prec.^a^		agglom.^b^ (183)	
PEG@POS–NH_2_	14	22.2–41.5	0.33	18.6	0.13	44	0.32
PEG@POS–NH_2_Q1	12	18.9	0.27	110	0.19	57	0.34
PEG@POS–NH_2_Q2	16	21.4	0.26	111	0.22	69	0.32

^a^”prec.” indicates that the sample precipitated macroscopically; ^b^”agglom.” indicates that agglomeration occurred and the agglomerates were nearly as big as the specified filter pore size of 450 nm; the corresponding mean radius values are given in parenthesis. In the other cases (radius values not in parenthesis) measurements were spot-checked for filtration loss by comparison with results obtained after filtration through filters of larger pore size (>2 μm).

**NexSil20:** DLS of NexSil20 yielded a hydrodynamic radius of 17.1 nm. The μ_2_ value of 0.11 indicates that NexSil20 is not monodisperse but still has a narrow size distribution. The measured hydrodynamic radius is marginally larger compared to TEM size determination. From these findings we conclude that there are a very small number of agglomerates present in the sample. Due to the *R^6^* dependence of the scattering intensity (see above), a small number of agglomerates have a comparably high impact on the mean value in DLS, whereas in TEM, one is not able to detect agglomerates due to the fact that the arrangement of the particles is influenced by the preparation.

Zeta potential (ZP) determination in water containing small amounts of salt (5 mM NaBr) yielded a value of −40 mV, which clearly shows the negative surface charge that is responsible for the colloidal stability of the dispersion. Generally, the absolute value of the zeta potential decreases under conditions of high salinity, which are predominant in cell culture medium. For example, for NexSil20, a zeta potential value of −20 mV is found. However, under conditions of physiological salinity, zeta potential determinations based on electrophoretic mobility measurements should be treated with great care. As they are influenced by a multitude of factors, such as surface charge, salinity and by the interactions that are present in the colloid, the applied models to derive zeta potential values from electrophoretic mobility become inadequate [77].

**POS-NH_2_:** The sample POS-NH_2_ yielded a hydrodynamic radius of 13.5 nm and a μ_2_ value of 0.11. After alkylation with ethyliodide or, alternatively, with 2-iodoethanol (POS-NH_2_Q1 and POS-NH_2_Q2, respectively) an increase of around 2 nm in hydrodynamic radius was observed. The distinctly higher μ_2_ values of 0.29 and 0.27 for the quaternized samples are presumably caused by a minor fraction of agglomerates. Agglomeration might be induced here by the change of the surface properties during the quaternization procedure. Thus, the higher μ_2_ values reveal a decrease in colloidal stability of the samples after quaternization. Zeta potential measurements show a rather constant value of approximately +30 mV in 5 mM NaBr for all three samples indicating that, at least under low salt conditions, the degree of quaternization does not influence the charge of the particles.

**Figure 2:**
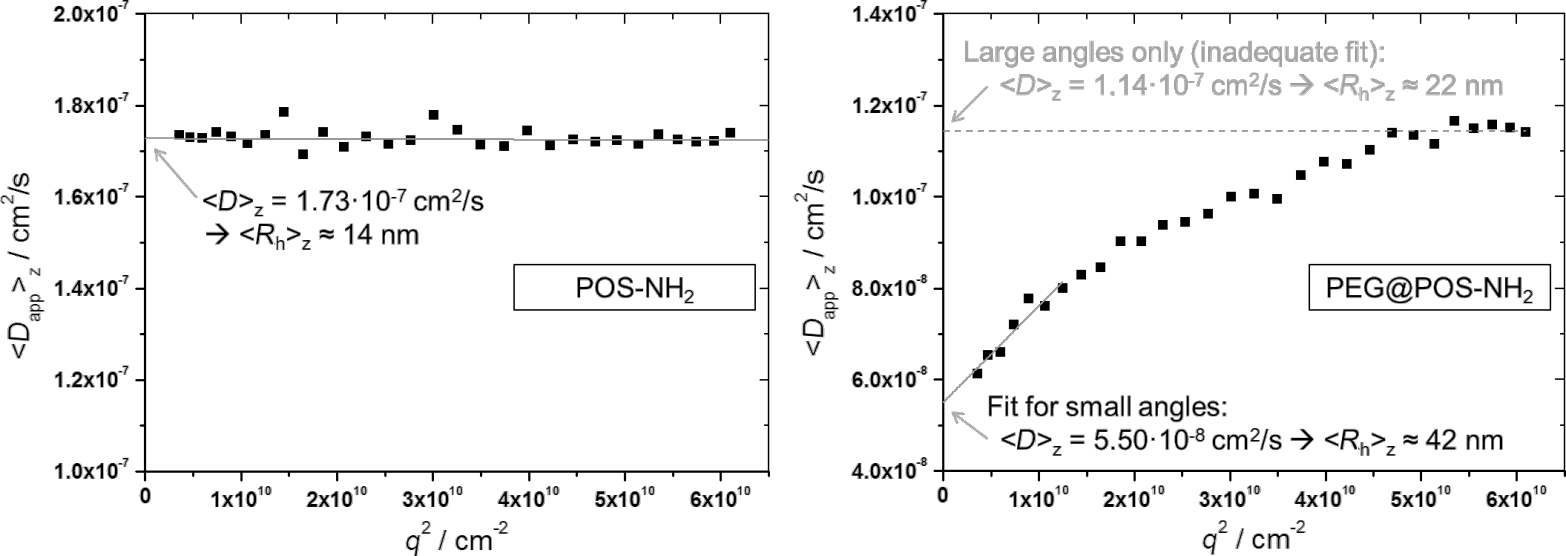
Apparent self-diffusion coefficients (*D*_s,app_) from angular-dependent DLS measurements with respect to the squared scattering vector (*q*^2^) for the samples POS-NH_2_ and PEG@POS-NH_2_. In contrast to POS-NH_2_, the sample PEG@POS-NH_2_ shows an angular dependence of the *D*_s,app_ values and a linear fit of only the large scattering angles (θ < 120°) would disregard a second fraction of larger particles. This is a clear indication of agglomeration and an explanation for the large polydispersity value of this sample. Furthermore, this example nicely illustrates that especially for polydisperse samples only the extrapolation *q^2^* → 0 can yield true values for *D*_s_ (and similarly also for *R*_h_).

**PEG@POS-NH_2_:** With the introduction of PEG, the mechanism of colloidal stabilization is changed towards steric stabilization and colloidal stability is expected to increase. However, the DLS measurement of the resulting sample PEG@POS-NH_2_ revealed a hydrodynamic radius of 41.5 nm and μ_2_ values of 0.33. Additionally, in contrast to the non-PEGylated sample POS-NH_2_, a significant dependence of the apparent diffusion coefficients on the scattering angle was observed for the sample PEG@POS-NH_2_. This is shown in Figure 2 in which the apparent self-diffusion coefficients, *D*_S,app_, are plotted against the (squared) scattering vector *q* (which correlates to the scattering angle) for these two samples. As already mentioned, a dependence of *D*_S,app_ on *q*^2^ is generally another indication of polydispersity (in addition to the μ_2_ value). As in the case of POS-NH_2_, for PEG@POS-NH_2_ this observation can also be correlated to agglomeration of a minor fraction of the particles. Although sterically stabilized colloids are generally assumed not to agglomerate, the polymer chains might entangle due to polymer–polymer and polymer–solvent interactions resulting in loose entanglements of the colloidal particles.

Quaternization of the remaining secondary amine groups of the PEGylated particles with ethyliodide or 2-iodoethanol leads to an increase of the inter-particle repulsive forces. Therefore, the samples PEG@POS-NH_2_Q1 and PEG@POS-NH_2_Q2, as expected, decreased in size. The measurement of the hydrodynamic radii of these samples yielded 18.9 nm and 21.4 nm, for PEG@POS-NH_2_Q1 and PEG@POS-NH_2_Q2, respectively. This decrease is caused by a loosening of the above described entanglements. In contrast to the observation of entanglements under non-physiological conditions, an improved stability could be verified under physiological conditions for the PEGylated samples, as described below. After PEGylation, the zeta potential drops from +30 mV to ca. +15 mV. The explanation for this observation is that the additional PEG layer leads to a shielding of the underlying positive charges.

#### Characterization under physiological conditions

A characteristic feature of biologically relevant media is a relatively high salinity which could have an impact on the colloidal stability of nanomaterials. As high salinity leads to a screening of surface charges, it diminishes electrostatic repulsion between NPs. In this context, the Debye-length scale is an important value describing the distance of electrostatic interactions between colloids. At physiological salt concentrations (at approximately 150 mM salinity), the Debye length is of the order of 0.8 nm (for symmetric electrolytes such as sodium chloride). This leads to coagulation of exclusively electrostatically stabilized nanoparticles in physiological media [78–80].

**NexSil20:** Table 1 shows the hydrodynamic radius of NexSil20 in presence of RPMI1640 cell medium as determined by DLS. A hydrodynamic radius of 17.6 nm was obtained in absence of proteins, which is in good agreement with the measurements in water with low salinity. The measurement was performed 15 minutes after sample preparation and repeated after 48 h (data not shown), not displaying any change of the hydrodynamic properties. Hence, the sample did not give evidence of agglomeration even under physiological salinity. The high stability of silica NPs can be attributed to a "gel-like" layer on the surface of the particles, which is discussed in the literature as sterically stabilizing coating [81].

With regard to in vivo and in vitro studies, NexSil20 was also characterized in the presence of serum proteins. RPMI1640 cell medium supplemented with fetal calf serum (FCS, 5 vol %) was applied as a model medium. DLS reveals an average hydrodynamic radius of 9 nm (and a μ_2_ value of 0.16) for the protein mixture, describing the presence of a large number of proteins with variable sizes. In contrast to the fact that the silica particles are sufficiently stabilized in the absence of biomolecules, these proteins induce the agglomeration of silica NPs yielding an average hydrodynamic radius of 91 nm for the agglomerates.

To derive agglomerate sizes in the cases in which proteins were present, DLS data were evaluated by the so-called multicomponent analysis [28]. By this procedure, the measured autocorrelation function is subdivided into single components with each component again expressed as a discrete autocorrelation function. As the autocorrelation functions of single components cannot be determined by a DLS measurement of the mixture of components, discrete correlation functions can only be obtained from measurements of the isolated substances. DLS data evaluation of the mixture of substances is then used to reveal the relative amplitudes of the single components as well as the size of the aggregate fraction.

**Figure 3:**
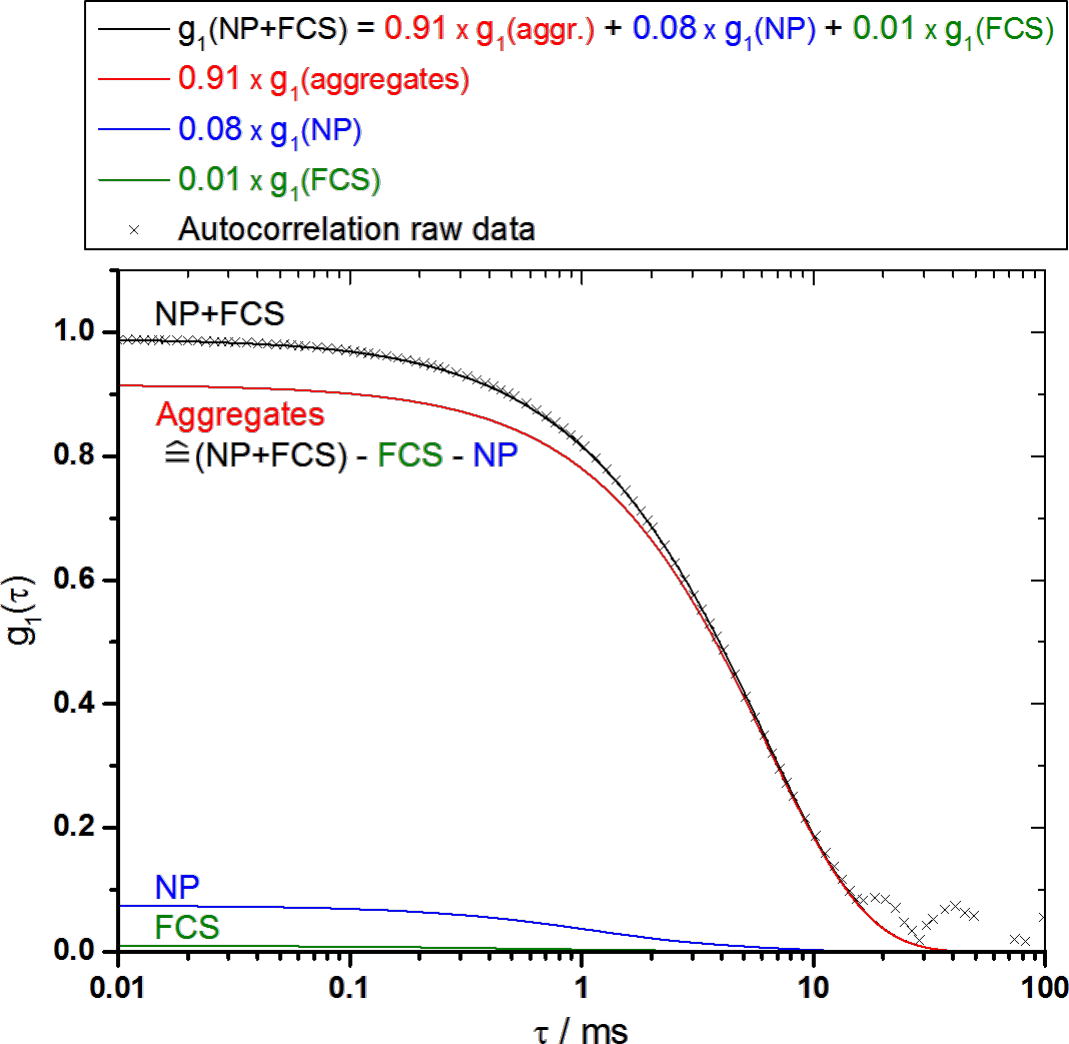
Multicomponent analysis [28] to evaluate the DLS measurement of the mixture of amorphous silica nanoparticles (NP) and serum proteins (FCS) at a scattering angle of 30°. As serum proteins (green), pristine nanoparticles (blue) and an additional fraction of agglomerates (red) contribute to the measured autocorrelation function g_1_(τ) (black crosses), the combined fitting function (black) also comprises contributions of these three components. The corresponding amplitudes are 0.01, 0.08 and 0.91, respectively.

As an example, the multicomponent analysis of the mixture of serum proteins (FCS), amorphous silica nanoparticles (NP) and the subsequently formed agglomerates at a scattering angle of 30° is shown in Figure 3. The autocorrelation function of pristine nanoparticles as well as that of the serum proteins were measured before and thus are preassigned. As result of the multicomponent analysis, the autocorrelation function of the mixture can be described as the autocorrelation function of serum proteins with an amplitude of 0.01 plus the autocorrelation function of pristine nanoparticles with an amplitude of 0.08 plus one additional component with an amplitude of 0.91, which can be assigned to agglomerates. The relaxation time of the aggregate component corresponds to a hydrodynamic radius of 91 nm.

As shown, the multicomponent analysis yields not only a size that can be assigned specifically to the aggregate fraction (in contrast to standard DLS data evaluation, which only yields a mean value), but it also allows a closer look at the contributions of the protein, particle and aggregate components to the scattering intensity. For the sample discussed above, this reveals that more than 90% of the scattered light at a scattering angle of 30° is caused by the agglomerates. The particle component contributes 8% and the protein component is almost negligible with an amplitude of less than 1%. The analysis of scattering intensity contributions for the other measurements that are described within this publication yielded comparable values. For larger agglomerates, the contribution of the agglomerate component reached even higher values in the range of 95%.

**Figure 4:**
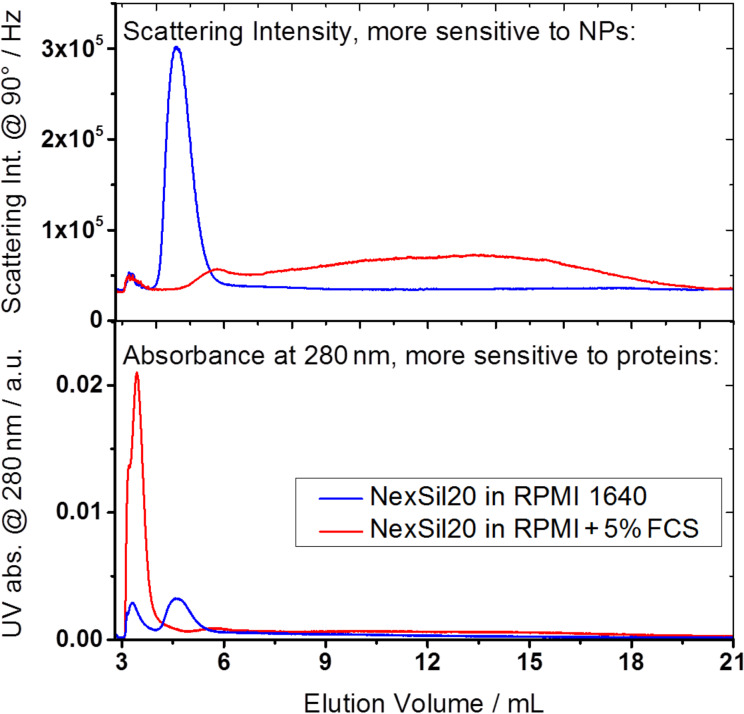
AF-FFF fractograms for NexSil20. Blue: NexSil20 prepared in RPMI cell medium; Red: NexSil20 prepared in RPMI cell medium in presence of 5 vol % FCS. The AF-FFF channel (ConSenxus) was operated with a cross flow of 2.5 mL/min and a detector flow of 1 mL/min, with a polysulfone membrane and a spacer of 190 μm thickness, and with an eluate concentration of 200 mg/mL NaN_3_ in deionized water.

Focusing not on the determination of absolute sizes but rather on a distribution analysis, AF-FFF was used for qualitative distribution analysis of the agglomerates. Figure 4 shows elugrams of NexSil20, prepared in RPMI cell medium as well as in RPMI + 5% FCS. In the upper graph, the scattering intensity signal at an angle of 90° is plotted. As derived by the multicomponent analysis, agglomerates can be detected very well by light scattering, exhibiting more than 90% of the total amplitude in the DLS experiment. In contrast, the scattering intensity of proteins was found to be negligible at the concentrations applied here. Therefore, UV absorbance at a wavelength of 280 nm was additionally measured because it provides a sensitive detection of proteins (lower graph).

In absence of serum proteins (blue lines) NexSil20 elutes as a sharp fraction indicating a narrow size distribution. Since scattering of light inside the UV absorbance cell distorts the absorbance detection, the silica particles cause an apparent UV absorbance signal despite the fact that they do not carry any chromophore groups. In the presence of serum proteins (red lines) a peak for the pristine NPs is observable by light scattering detection. This indicates that the single particles are not completely depleted. A shift in elution volume denotes the formation of the protein corona. Nevertheless, the use of this data for size determination would result in arbitrary results due to the fact that the surface properties are altered by protein adsorption. Additionally, a broad fraction was detected eluting at later elution volumes. Since in AF-FFF a sample is fractionated according to the hydrodynamic size in the order from small to large particles, this fraction corresponds to large agglomerates and it reveals a broad size distribution of the agglomerates. As UV absorbance at 280 nm is sensitive for proteins, the pronounced UV absorbance signal at the beginning of the elution process corresponds to the elution of the unbound proteins. This signal is part of the so-called void peak, which is an artifact of the method. The void peak represents non-fractioned components such as proteins, which are too small and elude from retention. Summarizing these observations, AF-FFF not only confirms the DLS results, but additionally yields a qualitative insight into the size distribution.

**POS-NH_2_:** As POS particles exhibit different surface properties compared to NexSil20, they also show different agglomeration behavior. The electrostatically stabilized samples POS-NH_2_, POS-NH_2_Q1 and POS-NH_2_Q2 macroscopically precipitated in RPMI cell medium due to an inefficient stabilization. During the sample preparation for DLS, the agglomerates were completely removed by the necessary sample filtration [26], therefore, a measurement was not possible. In the presence of serum proteins, the precipitation could not be observed by eye. Thus, the samples were filtrated after mixing and measured with DLS. The resulting hydrodynamic radius was 130 nm for the non-quaternized sample POS-NH_2_ and approximately 200 nm for the quaternized samples POS-NH_2_Q1 and POS-NH_2_Q2. In Table 1 these values are given in brackets since a loss of material due to the filtration process is likely (especially for the quaternized samples). Although the addition of serum proteins and the subsequent formation of a protein corona leads to an increase in colloidal stability of the electrostatically stabilized POS nanoparticles, in general, those particles offer poor colloidal stability under physiological conditions.

**PEG@POS-NH_2_:** In contrast to the electrostatically stabilized poly(organosiloxane) NPs, a hydrodynamic radius of 18.6 nm was detected for the sample PEG@POS-NH_2_ in RPMI medium. Additionally, this result and the μ_2_ value of 0.13 differs from the hydrodynamic size obtained in distilled water for this sample. In that case, the correlation function was superposed by polymer entanglements leading to loose agglomerates. Inter-particle polymer entanglements are mainly related to polymer–solvent interactions, as described by the Flory–Huggins parameter, and solvent quality changes with salinity due to the solvation shell around ions. As a qualitative observation, the entanglements of the sample PEG@POS-NH_2_ dissolve at physiological ion strengths. In the presence of proteins, agglomerates can be detected, however, they are much smaller in size than the electrostatically stabilized particles.

The quaternized and PEGylated samples PEG@POS-NH_2_Q1 and PEG@POS-NH_2_Q2 exhibit peculiar properties at physiological salinity. Analogous to the non-quaternized sterically stabilized sample, a hydrodynamic size similar to that of the pristine particles would be expected. However, radii of approximately 110 nm were measured, which suggests the formation of agglomerates or, since the particles are sterically stabilized, the formation of entanglements. In the presence of proteins, multicomponent analysis yields a hydrodynamic radius of 57 nm and 69 nm for the aggregate component. As these sizes are not superposed by the scattering signal of proteins, this observation gives evidence of a reduced diameter for the samples PEG@POS-NH_2_Q1 and PEG@POS-NH_2_Q2 in presence of proteins. For silver NPs, an additional sterical stabilization effect induced by protein adsorption was previously reported [82]. We assume that the stabilization of POS particles in the presence of proteins is driven by a similar stabilization mechanism.

### Summary of results

SiO_2_-based nanomaterials were characterized with respect to their size under non-physiological and physiological conditions by combining results from transmission electron microscopy, dynamic light scattering and asymmetric flow field-flow fractionation. Differences in the agglomeration state and therefore in the effective size of the materials were observed and related to surface properties. In particular, negatively charged silica nanoparticles were shown to be remarkably stable at physiological salinity, though unstable in the presence of proteins. In contrast, electrostatically stabilized poly(organosiloxane) particles with a positively charged surface macroscopically precipitated under physiological salinity. The presence of proteins inhibited the macroscopic precipitation, however, larger agglomerates still formed. The addition of a poly(ethylene glycol) coating onto the siloxane NPs yielded sufficient stability at higher ionic strengths. In presence of serum proteins, only small amounts of agglomerates could be detected. However, the alkylation of the secondary amine groups results in a significant growth of the hydrodynamic size at physiological salinity. In presence of serum proteins a dissipation of the agglomerates was observed. Thus, the increase in diameter in the absence of proteins is considered to be due to loose inter-particle entanglements of the polymer coating.

In summary, we demonstrated that the variation of the surface properties of SiO_2_-based nanoparticles directly affects the agglomeration behavior under non-physiological as well as physiological conditions. This behavior is particularly relevant for biomedical applications since it shows that particles that are precisely defined in size in deionized water or at low salt concentrations can become agglomerated and less defined under physiological conditions.

## Conclusion

The physiological impact of a nanomaterial is mainly determined by three factors. The first one is the question of how the nanomaterial interacts with biological barriers. Here, interactions with the alveolar surfactant film are of special interest, because it is the first biological barrier after exposure via the lung. Therefore, the mechanisms behind the penetration of hydrophobic siloxane NPs through this barrier were examined [71–73]. The second factor influencing the physiological impact of a nanomaterial is the formation of a protein corona. For silica NPs that exhibit amine and carboxy functionalities it could be shown that, depending on surface functionalities, the composition of the protein corona differs drastically [12]. The third factor is the influence of the protein corona-coated particle on interactions with the cellular membrane, and therefore, on cellular responses [14,83]. For example, negatively charged silica NPs were shown to exhibit a dose-dependent cytotoxicity, whereas PEGylated poly(organosiloxane) NPs do not reduce the cell viability [16].

As emphasized in the introductory part of the results section, verification of the nanomaterial properties in biologically relevant media is crucial to correlate the particle characteristics to the biological responses that these particles induce. With the work presented here, a well-characterized system of SiO_2_-based nanomaterials is exhibited which allows for variations in surface properties (surface charge and polarity) as well as in the mechanism of colloidal stabilization (electrostatically and sterically stabilized). Therefore, combining amine-functionalized siloxane NPs with silica NPs yields an appropriate model system to investigate how the surface properties of NPs influence the interactions of NPs with biological barriers, the formation of a protein corona and cellular responses.
